# 

**Published:** 1870-11

**Authors:** 


### CONTENTS:

Original Communications:

Art. I. Idiopathic Gangrena Genitalia in Infancy. By Jonathan W. Brooks, M.D. .... 641

Art. II. The Result of Twenty-two Doses of Chloral Hydrate; with Cases, Observations and Gleanings, at St. Luke's Hospital. By John E. Owens, M.D. .... 646

Art. III. Case from the Surgical Clinic of Prof. Moses Gunn, of Rush Medical College. Reported by H. F. Chesbrough, M.D. .... 653

Art. IV. Remarks on a Recently Discovered Mineral Spring at Portage, Mich. By E. Clapham, M.D. 656

Art. V. Bromide of Potassium in Leucorrhœa. By A. H. Kinnear, M.D. .... 661

Selections:

On the Value of a Large Supply of Food in Nervous Diseases .... 662

Importance of Proper Alimentation in Health and Disorders .... 669

Pamphlet Notices .... 675

Editorial .... 685

Cases in Cook Co. Hospital. Reported by C. T. Fenn, M.D. .... 690

Loot .... 694

Obituary, etc. .... 702

